# Detecting self-produced speech errors before and after articulation: an ERP investigation

**DOI:** 10.3389/fnhum.2013.00763

**Published:** 2013-11-11

**Authors:** Kevin M. Trewartha, Natalie A. Phillips

**Affiliations:** ^1^Centre for Neuroscience Studies, Queen's UniversityKingston ON, Canada; ^2^Centre for Research in Human DevelopmentMontreal, QC, Canada; ^3^Department of Psychology, Concordia UniversityMontreal, QC, Canada

**Keywords:** speech errors, speech production, event-related potentials, error-related negativity, conflict monitoring

## Abstract

It has been argued that speech production errors are monitored by the same neural system involved in monitoring other types of action errors. Behavioral evidence has shown that speech errors can be detected and corrected prior to articulation, yet the neural basis for such pre-articulatory speech error monitoring is poorly understood. The current study investigated speech error monitoring using a phoneme-substitution task known to elicit speech errors. Stimulus-locked event-related potential (ERP) analyses comparing correct and incorrect utterances were used to assess pre-articulatory error monitoring and response-locked ERP analyses were used to assess post-articulatory monitoring. Our novel finding in the stimulus-locked analysis revealed that words that ultimately led to a speech error were associated with a larger P2 component at midline sites (FCz, Cz, and CPz). This early positivity may reflect the detection of an error in speech formulation, or a predictive mechanism to signal the potential for an upcoming speech error. The data also revealed that general conflict monitoring mechanisms are involved during this task as both correct and incorrect responses elicited an anterior N2 component typically associated with conflict monitoring. The response-locked analyses corroborated previous observations that self-produced speech errors led to a fronto-central error-related negativity (ERN). These results demonstrate that speech errors can be detected prior to articulation, and that speech error monitoring relies on a central error monitoring mechanism.

## Introduction

Although speech production seems to be relatively effortless, speech errors occur that can often lead to embarrassment for the speaker; for example, addressing a law-enforcement official as *“Ociffer,”* rather than *“Officer.”* It has been estimated that we engage in revisions of speech errors during ~10% of our utterances (Nooteboom, 1980). Most theories of language production propose some form of a monitoring system that allows for the detection and ultimate correction of speech errors. However, there remains some debate about the exact nature of the monitoring mechanism(s) for self-produced speech errors (see Postma, 2000 for review). Recent findings have demonstrated that an electrophysiological correlate of error processing, called the error-related negativity (ERN), that is commonly observed in manual action errors (see Holroyd and Coles, 2002; Botvinick et al., 2004) is also elicited during overt speech errors in picture naming tasks (Ganushchak and Schiller, 2008a; Riès et al., 2011). This finding suggests that the same monitoring system involved in the detection of manual action errors is also involved in the detection of speech errors. However, it is unclear whether speech errors are monitored prior to, or only after speech formulation processes are complete. The first goal of the current study was to determine whether there are neurophysiological signatures of error monitoring during speech formulation, rather than after it is complete. The second goal was to determine if the ERN observed previously during picture naming also generalizes to other overt speech tasks such as the one employed here.

Models of speech production have a long history, and a review of those theories is beyond the scope of the current paper (e.g., Levelt et al., 1999; Postma, 2000). However, it is useful to briefly outline the various stages that are commonly proposed to underlie speech formulation and articulation. Speech production theories propose that speech formulation proceeds through a complex set of stages. Generally, speech production is thought to advance through three stages: the first is a conceptual stage that specifies the meaning and purpose of the pre-verbal message to be conveyed. The second stage consists of pre-articulatory (internal) speech during which the preverbal message is transformed into linguistic structure. This second stage requires lemma selection, syntactic framing, and phonemic specification such that a phonemic representation of the desired message can be passed to the third stage involving articulation during which the speech motor commands are generated and words are produced.

Speech production errors can take a variety of forms, and can occur at any stage of speech formulation (Postma, 2000). Regardless of how and when a speech error is generated, the error must be detected before it can be corrected. However, it is not clear exactly when errors are detected or what monitoring processes contribute to that detection. The perceptual-loop theory proposes that our own speech errors are monitored either at the level of the preverbal message (the conceptual loop), the phonemic representation (the inner loop), or after articulation has commenced (the external, auditory loop). The latter two types of monitoring are argued to proceed through the speech comprehension system that also allows us to detect the speech errors of others (e.g., Levelt, 1983; Levelt et al., 1999). Under this view, only the end products of each processing stage are monitored. Other theories, such as Node Structure Theory and production-based approaches, allow for errors to be monitored at various intermediate stages of speech formulation (e.g., Schlenck et al., 1987; MacKay, 1992). A recent approach to investigating the nature of speech error monitoring processes is to use electroencephalography (EEG) because those methods have been used extensively for investigating error detection during manual actions.

In tasks requiring manual responses, errors have been associated with a particular event-related potential (ERP) component characterized by a larger negative deflection in the EEG immediately following errors compared to correct responses. This so called ERN is observed over fronto-central electrode sites, peaks between 50 and 100 ms after the response, and has been localized to the anterior cingulate cortex (ACC) of the medial frontal lobes (for review see Holroyd and Coles, 2002). The ERN has been observed using a variety of different tasks including the flanker, Simon, Stroop, and go/no-go tasks, among others. It has also been observed during a variety of response modalities including button presses, arm movements, and saccades. Computational models and empirical data have suggested that the ERN likely reflects processing of response conflict, rather than error detection *per se* (e.g., Botvinick et al., 2004; Yeung et al., 2004). This view is supported by the observation of additional stimulus-locked ERP components associated with conflict (e.g., N2 and the N450) in the Stroop (e.g., West, 2004), go/no-go (e.g., Nieuwenhuis et al., 2003) and flanker tasks (Yeung et al., 2004). These components have a similar topography as the ERN and have also been localized to the ACC. Together these findings suggest that the ACC serves as a central monitor of stimulus and response conflict (Botvinick et al., 2004). As such, these components might prove to be useful indices of conflict monitoring during speech production. An important point for the current paper is that stimulus-locked ERP components provide evidence that action errors, or more specifically the potential for future errors, can be monitored prior to the execution of the actual response.

In speech tasks, response-locked analyses have demonstrated that errors made during covert speech tasks also lead to an ERN-like waveform. For example, in one paradigm, participants covertly named line drawings while making button presses if a certain phoneme was present in the name. Incorrect button presses (i.e., false alarms) in this modified go/no-go task led to an ERN (Ganushchak and Schiller, 2006, 2008b). These findings suggest that error detection during language tasks occurs via the same conflict monitoring system involved in action errors. However, the locus of the error in these studies is ambiguous since the ERN was observed following a button press rather than a vocal error. It remains unclear whether the error being detected is in the speech production process, or at the manual response selection stage. Evidence consistent with error detection during vocal responses has been observed for speech errors elicited in the Stroop task (Masaki et al., 2001). However, it is important to point out that ACC activity associated with conflict monitoring in the Stroop task has also been observed following manual, in addition to vocal responses (see Barch et al., 2001). As such, it is difficult to specify whether the ERN observed by Masaki and colleagues reflects monitoring of the vocal error itself, or of response conflict that is inherent in the task. Similar evidence comes from a study using a spoonerism task (e.g., Möller et al., 2007). Participants were shown inductor word pairs that started with the same two first letters (e.g., DUCK BILL; DUST BIN) and were then cued by a vocalization prompt to speak aloud only for target pairs (e.g., BARN DOOR). Trials in which an error was made led to an ERN-like waveform. These findings are informative, but given that the analyses were locked to the stimulus and to a vocalization prompt rather than the error response, one could question whether the observed ERN-like waveform can be definitively linked to the monitoring of the vocal error itself. Moreover, the spoonerism task can be thought of as a modified go/no-go task that may introduce response conflict via the lower probability of go compared to no-go trials. It has been shown previously that changing the proportion of go and no-go trials can modulate conflict-related ERP components (e.g., Nieuwenhuis et al., 2003). Thus, it cannot be ruled out that the error-related ERP modulation observed by Möller et al. (2007) could be due to general probability-related conflict, rather than speech errors. Nonetheless, these studies provide important support for the hypothesis that speech errors are monitored via a central conflict monitoring mechanism. However, stronger evidence would consist of a true response-locked ERN that is observed following vocal errors in tasks that are not confounded by other types of response conflict.

Recent research has provided such strong evidence. Two studies have shown that vocal errors elicited in a picture-naming task lead to an ERN akin to that observed following manual action errors (Ganushchak and Schiller, 2008a; Riès et al., 2011). In both studies participants were shown pictures of line drawings and asked to name them out loud. Ganushchak and Schiller (2008a) observed an ERN maximal over fronto-central electrode sites for errors but not correct responses. Riès et al. (2011) also observed an ERN following errors, but also for correct responses. The ERN on correct responses was smaller and exhibited an earlier peak than the ERN for errors. This finding is consistent with previous observations of an ERN on correct trials during manual actions (e.g., Falkenstein et al., 2000; Vidal et al., 2000; Bartholow et al., 2005). An ERN on correct trials has been used as evidence in support of the conflict monitoring interpretation of the ERN. Under this interpretation, errors represent a special case in which response conflict was unresolved, and a central monitoring mechanism detects this response conflict. The idea that the ERN following speech errors reflects response conflict monitoring is supported by a recent observation that an ERN-like component is sensitive to the extent to which multiple vocal responses are activated during a picture naming task (Acheson et al., 2012).

These studies provide clear support for the idea that speech errors are monitored by the same conflict monitoring system that monitors for non-vocal errors (Ganushchak and Schiller, 2008a; Riès et al., 2011). Moreover, the timing of the ERN provides important insight into the specific stage of speech formulation during which this monitoring takes place. In both studies, the ERN peaked approximately 50–100 ms after the initiation of the vocal response. This early timing indicates that the ERN does not reflect post-error processing via auditory feedback in an external loop. Rather, the ERN likely reflects the monitoring of a pre-articulatory error in speech formulation during the inner loop. It is reasonable to speculate that monitoring is occurring during the latest stages of internal speech formulation, perhaps even during the preparation of speech motor commands. At the very least, it seems likely that the ERN reflects evaluation of the end products of phonetic encoding as conceived by recent speech production models (e.g., Levelt et al., 1999). An important motivating question for the current study is whether speech errors can be monitored earlier in the speech formulation process, for example, during phonological encoding, lexical selection, or even concept formation.

There are a number of studies that have provided behavioral evidence for speech monitoring prior to the articulation stage. For example, Wheeldon and Levelt (1995) asked participants to listen to English words, and silently generate the Dutch translation and monitor for the occurrence of different word segments in that translation. The critical comparison is between trials on which the target segment was the first, compared to second syllable in the word. Participants took longer to respond when the target was the second syllable compared to the first. The most important finding for the current discussion was that the magnitude of this comparison did not change when participants were asked to concurrently count out loud while performing the monitoring task. This shows that even if the articulatory buffer is occupied participants were still able to perform the monitoring task in the same way. Thus, participants seemed to be monitoring some pre-articulatory representation of the target word. Similar behavioral evidence of pre-articulatory monitoring has been reported elsewhere, which largely informed the assumption that errors can be detected and repaired prior to articulation (see Postma and Kolk, 1993 for review). Given these behavioral findings, it should be possible to find neurophysiological evidence of error monitoring prior to articulation. However, to our knowledge, very few studies have taken this approach despite the fact that stimulus-locked analyses provide an important tool for understanding the temporal dynamics of pre-articulatory speech production processes (Riès et al., 2013). Of those studies that did, Möller et al. (2007) found evidence of an increased negativity on trials in which a spoonerism was made between 400 and 600 ms after stimulus presentation, prior to articulation. Also using a spoonerism task, Severens et al. (2011) recently reported that high conflict, taboo-eliciting trials were associated with an increased positivity 600 ms after stimulus presentation even though no overt error was made. This finding may be interpreted as evidence of a pre-articulatory correction of an error.

In the current study, we utilized stimulus-locked analyses to investigate ERPs associated with errors in speech formulation prior to articulation. We used a phoneme substitution task that is known to elicit spontaneous vocal errors (e.g., slips-of-the-tongue) on ~10% of trials (MacKay and James, 2004). The task involves visual presentation of single words containing target phonemes (e.g., /b/ or /p/). Upon encountering a word with a target phoneme (e.g., *RIPPED*), participants are required to mentally substitute the alternative target and vocalize the resulting word (e.g., *“RIBBED”*). This task was chosen because it requires participants to formulate a word by rapidly exchanging phonemes, and thus likely introduces conflict specifically related to the speech formulation process. That is, the task likely induces conflict between the target phoneme presented in the stimulus (e.g., /p/ in *RIPPED*), and the to-be-substituted phoneme (/b/ in *RIBBED*), rather than conflict between two fully prepared vocal responses (e.g., “*RIPPED”* vs. “*RIBBED”*). This differs from tasks like the Stroop task, where the conflict seems to arise between fully prepared, competing responses (see Szücs et al., 2009). This aspect of the task allows us to investigate ERPs associated with errors that occur during early stages of speech formulation, such as the phonological encoding of the internal speech stage that are necessary for performing the phoneme substitution. The present task also eliminates conflict associated with variability in response probability by requiring participants to vocalize a response on every trial. The experiment was also designed such that the direction of the substitutions between high and low frequency words was balanced in order to eliminate possible confounds associated with stimulus probability, pre-potent responses, and word frequency.

The studies reviewed above provide important information about the nature of both pre- and post-articulatory speech error monitoring, especially in specifying the role of a central error monitoring system in detecting errors after articulation has been initiated. However, the neurophysiological basis of speech error monitoring prior to articulation, by way of the conceptual and inner loops (Postma, 2000) has not been explored. The novel approach in the current study is to use both stimulus- and response-locked ERP analyses to investigate error monitoring before and after articulation has commenced in a single experiment. Speech errors were defined as mispronunciations of the target words during a phoneme substitution task. Stimulus-locked analyses were examined for evidence that trials that eventually lead to errors were distinguishable from those that were later error-free and thus might be an ERP signature of pre-articulatory monitoring. The phoneme substitution task induces a high level of conflict on every trial between the presented word and the to-be-produced word. According to conflict monitoring theory (e.g., Yeung et al., 2004) high-conflict trials that are correct should exhibit a larger N2 component than error trials. If speech errors prior to articulation are detected in a similar way to other action errors a similar pattern of N2 effects should be observed in the current stimulus-locked analyses. Response-locked analyses were conducted in order to confirm the presence of an ERN associated with vocal errors. The goal of this analysis was to investigate the generalizability of the ERN during speech production errors in a task other than the picture-naming task that has been previously used (Ganushchak and Schiller, 2008a; Riès et al., 2011). In order to corroborate previous evidence of an ERN following speech errors, we compared the amplitude over fronto-central sites between correct and incorrect trials immediately following the response, predicting that speech errors would elicit an ERN.

## Methods

### Participants

Sixteen undergraduate and graduate students (14 right handed) from Concordia University in Montreal, QC, Canada ranging in age from 19 to 35 years (*M* = 25.4, *SD* = 3.8) were recruited to participate in this study. All participants were native English speakers who gave informed consent to participate in the experiment. Concordia University's Human Research Ethics Committee approved this study.

### Materials and procedures

The design of this experiment was based on a modified word-substitution task used previously to elicit vocal errors (MacKay and James, 2004). In the current experiment, participants viewed individual words presented on the screen one-at-a-time for 80 ms. In this task, participants were asked to monitor for the occurrence of either a /b/ or a /p/ (e.g., *RIPPED)*, mentally substitute the alternative phoneme, and vocalize the resulting word (e.g., “*RIBBED”*) as quickly as possible. They were also instructed that if they observed a word that did not contain the target phonemes they should vocally respond with the word “NEITHER.” For the current purposes, the task used by MacKay and James (2004) was modified in order to increase the number of experimental trials by including an additional /d/ and /t/ substitution condition (e.g., seeing the word *TUSK*, and responding with *“DUSK”*).

All stimuli consisted of a suffix (optional) and a single syllable, and were presented in black, 34-point, Tahoma font on a computer monitor. Four different word lists were created (see Appendix): 88 word-pairs for the /b/ and /p/ substitution, 90 word-pairs for the /d/ and /t/ substitution, 50 words for a “read-only,” baseline condition, and 24 words used as catch trials (not containing the target phoneme) during the substitution blocks. An equal number of stimulus words required /b/ to /p/, and /p/ to /b/ substitutions. For the /b/ and /p/ substitution stimuli, words were selected that contained a /b/, or /p/ at the beginning, in the middle, or at the end of the word. Words were only included if a real English word could be created by substituting the /b/, for /p/ and vice versa. The /d/ and /t/ substitution stimuli were selected in the same way except with the phonemes /d/ and /t/ as the targets. The words used for the read-only condition and catch trials did not contain any of the target phonemes (i.e., /b/, /p/, /d/, or /t/). All word lists were matched for mean word length, frequency, as well as orthographic and phonographic neighbors (Balota et al., 2007). As an additional control, an equal number of items changed from high to low frequency (e.g., *BIT* to *PIT*), and vice versa (e.g., *LENT* to *LEND*) in the substitution conditions. Finally, a similar proportion of trials required a substitution from a voiced to unvoiced consonant, and vice versa. For half of the participants 52.5% of experimental trials required a voiced to unvoiced substitution, whereas for the other participants 47.5% of all trials required a voiced to unvoiced substitution. A practice list of 27 words was also created for each substitution type that consisted of English words that lead to pseudo-words after substitution. Participants saw only one of two counterbalanced versions of the experimental word lists, which were created as follows: the correctly substituted response words in list A were used as the stimulus words in list B, and vice versa.

All participants proceeded through the following order of conditions: the first block of 50 trials was used as a baseline measure to ensure that participants could correctly read and process the words during the brief presentation. For this block of trials participants were simply asked to read the words aloud as quickly as possible. Following the vocal response, there was an inter-trial interval of 200 ms before the next word was presented. The experimental trials began with 27 practice /b/ and /p/ substitution stimuli. After a short break, participants completed 88 /b/ and /p/ substitutions with 12 randomly placed catch trials, divided into two blocks. Finally, participants completed 27 practice trials using a /d/ and /t/ substitution rule, followed by 90 /d/ and /t/ substitutions with 12 random catch trials in two experimental blocks. No performance feedback was given during any stage of the experiment.

### Apparatus and electroencephalogram (EEG) recordings

For the current experiment we used Inquisit 3.0.4.0 (Millisecond Sofware LLC. Seatle, WA) to present words on the screen and to record vocal responses as individual.wav files. A standard headset microphone was used to record those vocal responses. A second microphone was used along with a custom built vocal triggering system that signaled the onset of the vocal responses using an amplitude threshold^1^.

The EEG acquisition software accepted those triggers and implanted codes in the EEG data stream for response synchronization. A continuous EEG was recorded with an active electrode EEG system, ActiveTwo (BioSemi, Amsterdam, NL), using a 64-electrode nylon cap, sampled at 512 Hz in a DC to 104 Hz bandwidth. All EEG data were re-referenced offline to the linked earlobes, and also filtered offline for frequencies between 0.1 and 50 Hz. Horizontal and vertical electrooculograms (HEOG and VEOG) were used to monitor eye movements and trials with HEOG activity exceeding +/−50 μV were rejected. Any excessive VEOG artefacts (i.e., eye blinks) were corrected using a technique equivalent to spatial principal components analysis (PCA) without rotation of components (the spatial filter correction technique, Method 2, NeuroScan Edit 4.3 manual, 2003). Trials with EEG activity and other motion artefacts exceeding +/−100 μV were rejected.

### Data analysis

While participants performed the task the first author monitored their vocal responses for errors by marking the trial number for any response that sounded like an error. All trials were re-checked for errors offline using the individual.wav files generated for each response, and were classified using criteria described by MacKay and James (2004). Briefly, nine different error categories were included (see Table 1 for categories, examples of errors, and relative frequencies). Omission errors were any words in which a segment was omitted during articulation (e.g., saying *pan*, instead of *pans*), whereas additions were words that included a new segment that should not have been uttered (e.g., saying *clups*, instead of *cups*). Participants also made substitution errors in which a phoneme substitution was made, but incorrectly. Sequential substitution errors were those responses in which the correct phoneme was inserted, but in the wrong place in the word (e.g., saying *baps*, instead of *labs* for the stimulus *LAPS*). Non-sequential substitution errors were words in which an incorrect phoneme was inserted into the word (e.g., saying *puck* instead of *pug* for the stimulus *BUG*). Non-substitution errors were trials in which the participant repeated the word they read, rather than making a substitution. Non-identification errors were trials on which participants said “*neither*” to an experimental word that contained a target phoneme. Fluency errors included stutters, false-starts, and “*uh*”s. Multiple errors were any trials that combined more than one of the above mentioned errors. Finally, error corrections were trials in which the participant spontaneously, and immediately corrected an initial error. Reaction time was calculated from stimulus onset to vocal response trigger.

**Table 1:** **Error types with absolute and relative frequencies in experimental trials**.

**Error type**	**Correct response**	**Error produced**	**Total number of errors mean (*SD*)**	**Absolute frequency (% of experimental trials) mean (*SD*)**	**Relative frequency (% of errors) mean (*SD*)**
Omission	Ripped Trips	“Rip” “Tips”	0.67 (0.90)	0.44 (0.48)	4.5 (5.2)
Addition	Bound Fat	“Bounds” “Flat”	0.86 (0.74)	0.56 (0.51)	7.1 (9.0)
Sequential substitution	Big Dub	“Pib” “Tud”	0.86 (1.19)	0.44 (0.60)	3.4 (4.5)
Non-sequential Substitution	Peck Dabs	“Beg” “Taps”	3.27 (5.05)	1.93 (2.80)	14.6 (13.0)
Non-substitution	Braised Hit	“Praised” “Hid”	1.33 (1.45)	0.56 (0.59)	6.5 (9.0)
Non-identification	Peach Rode	“Neither” “Neither”	0.60 (0.99)	0.30 (0.55)	2.9 (6.1)
Fluency error	Bugs Droops	“Ba-bugs” “Droo-droops”	1.53 (1.51)	1.04 (0.78)	13.3 (11.9)
Multiple errors	Swabbed Mate	“Swap” “Mad-mate”	5.93 (5.56)	3.44 (3.10)	31.4 (26.8)
Error correction	Crabs Hoots	“Crap- Crabs” “Hu- uh Hoots”	3.53 (4.10)	1.70 (2.20)	16.3 (16.6)

An important challenge for researchers exploring ERP components during overt speech production is the known contamination of the EEG signal by movements of the speech musculature (e.g., Brooker and Donald, 1980). This contamination is especially problematic for response-locked analyses. However, recent experiments have successfully explored ERP components during overt speech (e.g., Ford et al., 2001; Heinks-Maldonado et al., 2005; Hawco et al., 2009) and some researchers have employed statistical methods of removing muscle artifacts from the EEG signal (e.g., de Vos et al., 2010). Such methods were not employed in the current study as the contaminating effects of overt speech was minimized by requiring the production of only short, monosyllabic words that are uttered in isolation rather than in the context of a sentence. Moreover, any potential contamination in the response-locked signals obtained in this study will be the same for both errors and correct responses as the vocal nature of the response is the same in both cases. Finally, there is evidence that the central electrode site Cz that is most associated with error monitoring is not contaminated to the same extent by speech-related muscle movements as other electrode sites (Brooker and Donald, 1980). Consistent with this finding, we did not observe any significant EEG contamination at the midline electrode sites of interest in our analyses.

ERP analyses were conducted using Scan (software by Compumedics Neuroscan, Charlotte, NC, USA). Stimulus-locked epochs of 700 ms (−100 to 600 ms) were obtained to assess waveforms difference between stimuli that ultimately led to a correct vocalization and those that led to a vocal error. We focused our analysis on the central midline sites (FCz, Cz, and CPz) as it is commonly at these locations that conflict-related ERP components are observed during stimulus-locked analyses in manual action tasks.

Response-locked epochs of 500 ms (−300 to 200 ms) were obtained to assess waveform differences between correct and incorrect trials immediately following the response initiation. This epoch was divided equally into 50 ms intervals, and an ERN was assessed over the fronto-central sites FCz and CZ, between 50 and 100 ms after the response (Holroyd and Coles, 2002). This interval was compared with a 50 ms baseline interval preceding the response. The average amplitude for each interval was averaged across correct and incorrect trials and aggregated across participants. For all analyses, averages were baseline corrected to a 0 μV average of the 100 ms pre-stimulus, or pre-response interval. One participant was excluded from the ERP analyses because only one error trial survived artefact rejection.

## Results

The Greenhouse-Geisser non-sphericity correction (Greenhouse and Geisser, 1959) was applied to all repeated-measures ANOVAs with more than one degree of freedom (*df*) in the numerator. For each statistical comparison, uncorrected degrees of freedom, mean square error (*MSE*), partial eta squared (η^2^_*p*_), and adjusted *p*-values are reported. Analyses of simple effects are also reported following all significant main effects and interactions using the Bonferroni method. All statistical comparisons are considered significant at the α = 0.05 level unless otherwise stated.

### Behavioral results

We first verified that the brief presentation time was adequate for participants to process the words in the read-only condition and compared those responses to the two substitution conditions. A One-Way ANOVA comparing read-only to /b/ and /p/, and /d/ and /t/ substitution trials revealed a main effect for both error rate, *F*_(14,2)_ = 20.5, *MSE* = 415.3, η^2^_*p*_ = 0.59, *p* < 0.001, and reaction time, *F*_(14,2)_ = 38.2, *MSE* = 364, 996.9, η^2^_*p*_ = 0.72, *p* < 0.001. On average, participants only made errors on 1.9% of read-only trials (*SD* = 0.42%) and had an average reaction time of 272.0 ms (*SD* = 39.2) for correctly read words. Participants made significantly more errors on the /b/ and /p/ substitutions [*M* = 10.9%, *SD* = 7.6%, *t*_(15)_ = −5.3, *p* < 0.001], and /d/ and /t/ substitutions [*M* = 10.6%, *SD* = 5.6%, *t*_(15)_ = −6.9, *p* < 0.001], compared to the read-only trials. Also, the reaction time for the /b/ and /p/ [*M* = 471.0 ms, *SD* = 131.1 ms, *t*_(15)_ = −6.2, *p* < 0.001], and /d/ and /t/ substitutions [*M* = 472.7 ms, *SD* = 127.0 ms, *t*_(15)_ = −6.6, *p* < 0.001], were significantly longer than the read-only trials. The substitution trial types did not differ from one another either in error rate, *t*_(15)_ = 0.5, *p* = 0.62, or reaction time, *t*_(15)_ = −0.2, *p* = 0.88. Thus, for all subsequent analyses the substitution trial types were collapsed together. Overall, participants made errors on an average of 10.6% of substitution trials (*SD* = 6.1%) with an average reaction time of 472.0 ms (*SD* = 128.1 ms) for correct trials. Although slightly longer, the average reaction time for errors in the substitution trials (*M* = 500.1 ms, *SD* = 127.0 ms) did not differ significantly from correct trials, *t*_(15)_ = −1.9, *p* = 0.07.

Given that speech errors can take a variety of forms, it is important to provide additional details about the types of errors that were elicited in this experiment. To this end, we classified all errors into nine categories developed previously (e.g., MacKay and James, 2004). The relative frequencies of errors in each of the nine categories are summarized in Table 1. The most prevalent errors elicited in the current study were errors that included a combination of errors (i.e., multiple errors), error corrections, fluency errors, and non-sequential substitution errors. These four error types made up 75.5% of all errors elicited. The current study was focused on investigating electrophysiological correlates of pre-articulatory speech error detection (i.e., well prior to articulation). The categories least likely to represent errors in speech formulation processes are the non-substitution and non-identification errors, as these two categories reflect errors in performing the task, rather than speech errors *per se*. The remaining seven categories account for 90.5% of all errors, and represent errors in which it is likely that some aspect of early speech formulation, such as phonological encoding, went awry. For these reasons, non-transformation, and non-identification errors were eliminated from the ERP analyses. Unfortunately, because an insufficient number of error trials were elicited in each of the remaining error categories, fine-grained ERP analyses comparing the different error types was not possible. Thus, as has been done previously, all error types were pooled for the ERP analyses (e.g., Riès et al., 2011).

### Stimulus-locked ERP analysis

**Figure 1:**
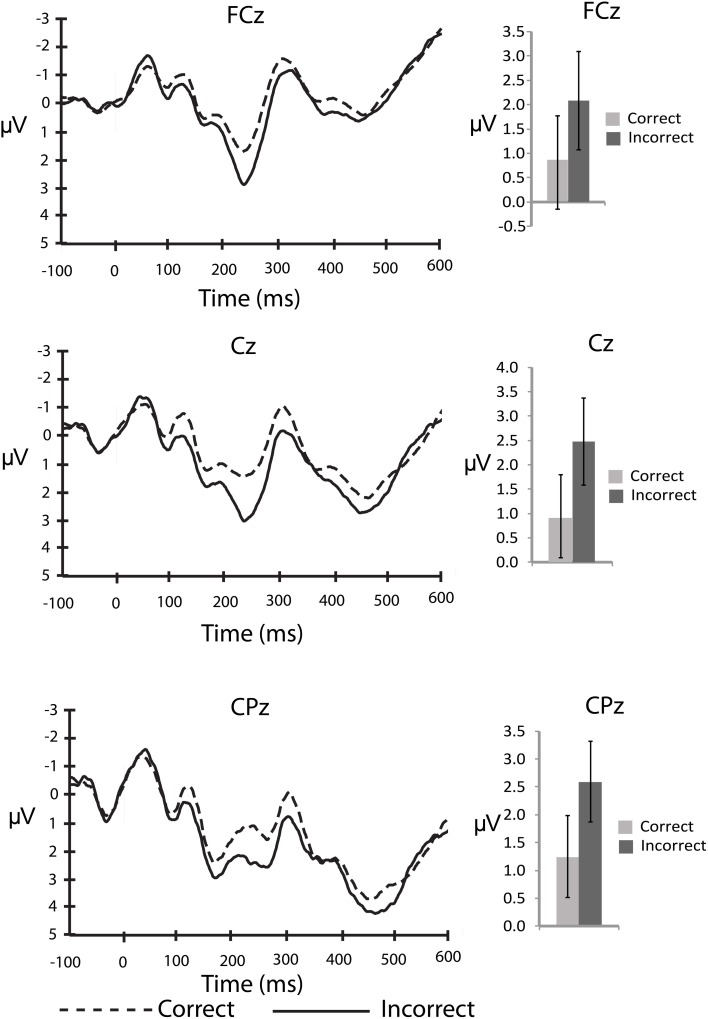
**Stimulus-locked, grand averaged waveforms for electrode sites: FCz, Cz, and CPz**. Figure shows the average waveform for correct (dashed line) and incorrect (solid line) responses (panels on left side). The P2 component can be observed between 200 and 275 ms post-stimulus. The panels on the right show the averaged amplitudes within the P2 window, with standard error bars. The N2 can be observed between 275 and 375 ms post-stimulus.

An important question about the nature of error monitoring during speech production is whether errors are detected prior to articulation. To explore this possibility with the current data we compared the averaged amplitude of the ERP signal following the stimuli that ultimately led to a correct, vs. incorrect vocalization. For the stimulus-locked analyses, the number of error trials that survived artefact rejection ranged from 5 to 30 trials^2^ (*M* = 14.5, *SD* = 7.2) whereas the number of correct trials included in the averages ranged from 49 to 162 trials (*M* = 113.4, *SD* = 27.9). This average is well above a recent report of the minimum number of trials needed for a stable ERN (Pontifex et al., 2010). By visual inspection of the stimulus-locked waveform (Figure 1) it is clear that there are early differences between stimuli that led to correct and incorrect responses. A P2-like, positive peak appears larger for incorrect compared to correct responses. In order to explore this difference we compared the average amplitude of the waveform between 200 and 275 ms post-stimulus to characterize the P2. This difference between correct and incorrect responses was compared using 2 (response type) × 3 (electrode site) ANOVA for the P2 interval. There was a significant main effect of response type, *F*_(13,2)_ = 5.0, *MSE* = 8.4, η^2^_*p*_ = 0.26, *p* < 0.05 such that the P2 was larger for stimuli that led to errors compared to those that led to correct responses. There was no effect of electrode site (*p* > 0.76), and no interaction between response type and electrode site (*p* > 0.57).

Overall, early stimulus-related processing associated with an upcoming error is evidenced by the P2 waveform differences over fronto-central electrode sites. Previous work has associated the P2 with orthographic neighborhood density (e.g., Taler and Phillips, 2007). To test whether the observed P2 amplitude differences in this study could reflect orthographic neighborhood density effects we assessed the orthographic, and phonological neighborhood density of words that led to speech errors compared to words that led to correct substitutions. Interestingly, the average orthographic and phonological neighborhood density ratings of words that led to an error (*M* = 11.1, *SD* = 1.3, and *M* = 20.9, *SD* = 3.4, respectively) were lower than the ratings of words that led to correct responses (*M* = 12.5, *SD* = 0.2, and *M* = 23.9, *SD* = 0.4, respectively). *T*-test comparisons between errors and correct trials indicated that these differences were significant for orthographic, *t*_(14)_ = 3.86, *p* < 0.01, and phonological neighborhood density ratings, *t*_(14)_ = 3.16, *p* < 0.01. This finding supports previous work linking the P2 to orthographic and phonological processing during word recognition. Also, words that led to speech errors had lower word frequency ratings (*M* = 51.7, *SD* = 84.6) than words that led to correct responses (*M* = 102.3, *SD* = 12.1). This difference was marginally significant, *t*_(14)_ = 2.11, *p* = 0.053.

Given that words that ultimately led to an error differed in neighborhood density and word frequency, the P2 amplitude differences observed between incorrect and correct responses could reflect differences in word recognition processes rather than error processing. To test this potential confound we conducted a median split on neighborhood density and word frequency for words that led to correct responses (note that there was not a sufficient number of trials to conduct such an analysis on errors). If the P2 differences that we observed are purely the result of neighborhood density or word frequency effects, words that are high on either of those ratings should be associated with a larger P2 component. To this end, we compared the averaged amplitude of the waveforms from 200 to 275 ms post-stimulus for words that were above and below the median neighborhood density in a density rating (high vs. low) by electrode site (FCz, Cz, and CPz) ANOVA. The mean amplitude for high neighborhood density words (*M* = 1.38, *SE* = 0.87) did not differ from low neighborhood density words (*M* = 1.27, *SE* = 0.91). There were no main effects of density rating (*p* = 0.79) or electrode site (*p* = 0.66), and no interaction (*p* = 0.10). We also compared the averaged amplitude of the waveforms between 200 and 275 ms post-stimulus for words that were above and below the median word frequency rating for correct responses in a frequency rating (high vs. low) by electrode site (FCz, Cz, and CPz) ANOVA. Again, there were no differences in P2 amplitude between high (*M* = 1.36, *SE* = 0.90) and low frequency words (*M* = 1.48, *SE* = 0.89) at any electrode site. There were no main effects of word frequency (*p* = 0.74) or electrode site (*p* = 0.74), and no interaction (*p* = 0.80). Thus, the fact that the amplitude of the P2 was not modulated by neighborhood density or word frequency ratings suggests that the error-related P2 amplitude increase cannot be accounted for by differences in stimulus characteristics. Instead, the increased P2 amplitude for errors likely reflects a neurophysiological signature of an error occurring prior to articulation.

It is also clear from visual inspection of Figure 1 that both correct and incorrect responses elicited an N2 component of similar amplitude. To verify that a robust N2 component was elicited in this experiment we quantified the magnitude of the N2 component by calculating a peak to trough difference score. We calculated the difference between the peak of the P2 between 200 and 275 ms post-stimulus and the peak of the N2 between 275 and 375 ms post-stimulus for each individual and compared the average of this N2 magnitude to zero for both correct and incorrect responses in separate *t*-tests at each midline electrode site (FCz, Cz, and CPz). At all three electrode sites the N2 magnitude was significantly greater than zero for both correct and incorrect responses after using a Bonferroni correction for multiple comparisons (Table 2). This finding indicates that a high level of response conflict was induced during all trials in this paradigm.

**Table 2:** **Comparing N2 magnitude to zero for words that led to correct and incorrect responses**.

**Electrode site**	**Response accuracy**	**Mean (*SD*)**	***T*-test**
FCz	Correct	−7.81 (2.75)	*t*_(14)_ = −11.02, *p* < 0.001
	Error	−10.91 (3.13)	*t*_(14)_ = −13.49, *p* < 0.001
Cz	Correct	−7.65 (2.88)	*t*_(14)_ = −10.30, *p* < 0.001
	Error	−10.78 (3.93)	*t*_(14)_ = −10.64, *p* < 0.001
CPz	Correct	−7.94 (3.17)	*t*_(14)_ = −9.70, *p* < 0.001
	Error	−10.73 (4.55)	*t*_(14)_ = −9.14, *p* < 0.001

T-tests are considered significant at Bonferroni corrected p-value of 0.008.

Our initial hypothesis was that if conflict monitoring resources are recruited to detect pre-articulatory speech errors, the N2 should differ between correct and incorrect responses. To determine if this was the case we conducted a 3 (electrode site) × 2 (response type) ANOVA for the 100 ms interval between 275 and 375 ms post-stimulus to characterize the N2 component. The main effect of response type was not significant (*p* > 0.60) suggesting that there were no reliable differences between stimuli that led to correct responses compared to incorrect responses at any of the electrode sites. There was a significant main effect of electrode site, *F*_(13,2)_ = 5.0, *MSE* = 8.4, η^2^_*p*_ = 0.44, *p* < 0.05, such that the N2 amplitude was more negative at FCz than Cz (*p* < 0.05) and CPz (*p* < 0.05). There was also no interaction between electrode site and response type (*p* > 0.60). The lack of error-related N2 amplitude difference demonstrates that the stimuli that ultimately led to an incorrect response did not recruit conflict monitoring resources any more than those that led to a correct response.

### Response-locked ERP analysis

**Figure 2:**
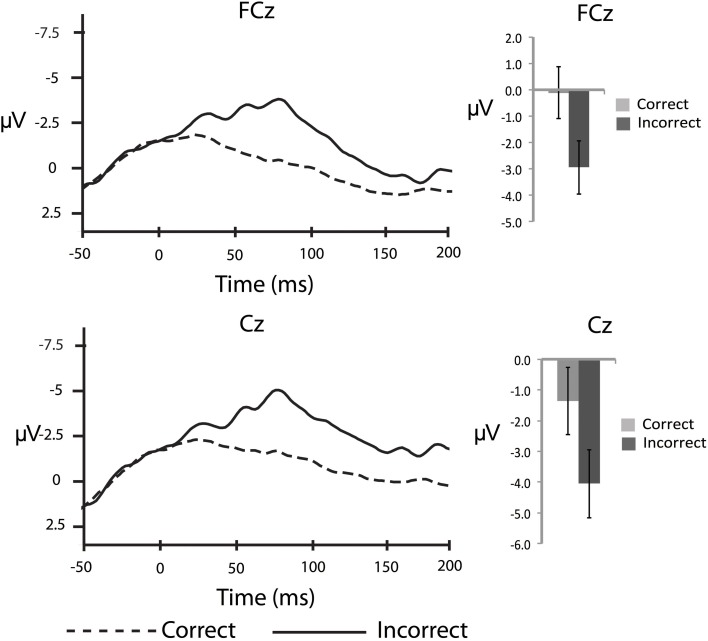
**Response-locked, grand averaged waveforms for fronto-central electrode sites: FCz and Cz**. Figure shows the average waveform for correct (dashed line) and incorrect (solid line) responses (panels on the left side). The ERN can be observed after the response, peaking at ~75 ms post-error response. The panels on the right side show the averaged amplitudes within the 50–100 ms post-response interval, with standard error bars.

In order to corroborate previous observations that speech errors elicit an ERN similar to the ERN observed for other types of action errors, we also conducted response-locked analyses. To characterize the ERN we compared the averaged amplitude in the 50–100 ms interval after the response between correct and incorrect responses in the substitution trials over the fronto-central electrode sites: FCz and Cz (e.g., Holroyd and Coles, 2002; Yeung et al., 2004). As a baseline comparison, the averaged amplitude in the 50 ms interval immediately before the response was compared between correct and incorrect responses. For the response-locked analyses, the number of error trials that survived artefact rejection ranged from 5 to 32 trials (*M* = 14.0, *SD* = 7.7) whereas the number of correct trials included in the averages ranged from 61 to 166 trials (*M* = 122.0, *SD* = 35.9). These data were subjected to separate 2 (electrode site) × 2 (response type) ANOVAs. In the 50–100 ms interval there was a significant main effect of response accuracy, *F*_(14,1)_ = 9.9, *MSE* = 11.1, η^2^_*p*_ = 0.41, *p* < 0.05, such that there was a larger negative waveform for incorrect compared to correct responses over both electrode sites (Figure 2). The comparison between correct and incorrect responses was not significant during the 50 ms interval prior to the response [*F*_(14,1)_ = 1.0, *MSE* = 0.008, η^2^_*p*_ = 0.07, *p* = 0.34]. This finding confirms the prediction that an ERN with typical topography and latency would be associated with self-produced speech errors.

## Discussion

The goals of the current study were to investigate electrophysiological correlates of both pre- and post-articulatory error monitoring during speech production. To this end, we employed a phoneme substitution task that is known to elicit self-produced speech errors (MacKay and James, 2004) and analyzed stimulus- and response-locked ERP waveforms following correct and incorrect vocal responses. Stimulus-locked analyses revealed that stimuli for which participants failed to correctly substitute the required phoneme did not elicit increased recruitment of general conflict monitoring resources, at least as measured by the N2 component. The N2 did not differ between stimuli that led to errors compared to correct responses. However, ERPs elicited by the presentation of words during substitution trials that led to speech errors were distinguishable from those that did not lead to speech errors by a larger P2 component. This observation suggests that the P2 represents a neurophysiological signature of errors that occur during speech formulation, prior to articulation. The response-locked analysis confirmed our hypothesis that speech errors would be followed by an ERN over fronto-central electrode sites. These findings corroborate and extend previous research that posits a role for the ACC in self-produced speech monitoring and are discussed below in terms of current speech production models.

Our initial hypothesis was that if speech errors are monitored prior to articulation a neurophysiological signature of error monitoring should be observed in our stimulus-locked analyses. In paradigms that require manual actions, it is the N2 component that is typically associated with conflict monitoring processes that are engaged prior to the overt action (e.g., Yeung et al., 2004; Carter and van Veen, 2007; Randall and Smith, 2011; Smith, 2011). Theories of speech production have proposed that speech errors are detected by the same central conflict monitoring mechanisms recruited for manual actions (Ganushchak and Schiller, 2008a; Riès et al., 2011). On this basis, our prediction was that stimuli that ultimately led to a correct response would be preceded by a larger N2 than stimuli that led to an error. The current results do not support this hypothesis. Instead, both correct and incorrect responses were associated with an N2 component of equal amplitude suggesting that participants were always monitoring for conflict during early stages of the speech formulation processes. The substitution task introduces a high level of conflict between the visually presented word and the to-be-produced word. In this sense it is likely that conflict monitoring processes are recruited on every trial. This finding could be interpreted as evidence that conflict monitoring resources are recruited during speech tasks that involve response conflict. Notably, the N2 component has been observed in a variety of experimental conditions, many of which do not involve response conflict (see Folstein and van Petten, 2008). The N2 component can be divided into numerous sub-components based on topography and eliciting conditions. The N2 in the current data is largest at more anterior than posterior sites. The anterior N2 has been associated specifically with novelty processing and visual mismatch in tasks like the oddball paradigm, and cognitive control processes in tasks like the flanker task and the go/no-go task. It seems unlikely that the current N2 reflects stimulus novelty or visual mismatch as stimuli were carefully matched on word length, frequency, and neighborhood density, and an equal number of trials required /b/, /p/, /d/, and /t/ substitutions. A more likely interpretation is that the phoneme substitution task requires recruitment of cognitive control processes such as performance monitoring on every trial. However, further research will be required to specify the role of conflict monitoring processes during speech formulation prior to articulation.

Although the observation of an N2 component on all trials suggests that general conflict monitoring resources are recruited during the task, the N2 does not appear to specifically reflect early speech error detection. However, the stimulus-locked analysis did distinguish words that led to correct responses from those that led to incorrect responses. Specifically, there was an increase in the amplitude of the P2 waveform following stimuli that led to an incorrect, compared to correct response. This observation is consistent with the idea that errors can be monitored prior to articulation. Given the timing of the P2 component it is clear that this monitoring is occurring during an early stage of speech formulation. Previous research has associated the P2 with early orthographic processing during word recognition. For example, the amplitude of the P2 has been shown to be larger when participants recognize words that have a high, compared to low neighborhood density rating (e.g., Taler and Phillips, 2007). The P2 component is also similar to the recognition potential, an ERP component associated with viewing recognizable stimuli such as pictures and words (see Martín-Loeches, 2007 for a review). This component is sensitive to orthographic characteristics of word stimuli (e.g., Martín-Loeches et al., 1999), and has largely been attributed to lexical selection processes (e.g., Hinojosa et al., 2001a,b). These findings suggest that the P2 may reflect initial access to, or activation of lexical candidates sharing orthographic features with the presented word. In the context of speech production models (see Postma, 2000), a larger P2 component for error trials would place the monitoring process in the internal speech stage, potentially during formation of the phonemic representation of the to-be-spoken word.

Given that the P2 component is sensitive to the orthographic neighborhood density of the stimulus word, it could be argued that the larger P2 for errors in the current data is specifically related to neighborhood density effects rather than error monitoring *per se*. In word recognition, early visual processing leads to the activation of a subset of compatible lexical entries in the mental lexicon. During lexical selection, the appropriate candidate must be selected from this pool of activated compatible lexical entries. Thus, phonological and orthographic neighbors are likely activated upon encountering a visual word stimulus. In the phoneme substitution task employed in the current study, the target, to-be-produced word is one of those activated orthographic neighbors. As such, it could be predicted that words with high neighborhood density should lead to increased competition between the many activated lexical candidates, resulting in a larger P2, and leading to higher error rates. The current data do not support this prediction. Instead, words that led to an error had lower neighborhood density ratings, but a larger P2 component, while words that led to correct responses had higher neighborhood density ratings and a smaller P2. This pattern is not consistent with a neighborhood density interpretation of the P2 differences between correct and incorrect trials. In fact, our data suggest that the activation of many orthographic neighbors of the presented words facilitates the ultimate production of the neighbor containing the appropriate phoneme substitution. This interpretation is consistent with behavioral evidence that lexical decisions and word naming are faster for high compared to low neighborhood density words (e.g., Sears et al., 1995; Forster and Shen, 1996; Carreiras et al., 1997).

Further evidence against a neighborhood density explanation of the current P2 effects comes from a direct comparison of waveforms elicited by words with high vs. low neighborhood density. For all trials that led to a correct response we conducted a median split based on orthographic neighborhood density ratings to determine if the amplitude of the P2 component was sensitive to neighborhood density in the phoneme substitution task. The data revealed that the size of the P2 component did not vary as a function of neighborhood density. Moreover, a similar comparison of words with high vs. low frequency failed to reveal any differences in the amplitude of the P2 component. The P2 amplitude was only sensitive to speech errors in the current experiment, providing additional support for an error monitoring explanation of the increased P2 amplitude.

Nonetheless, it seems likely that neighborhood density plays a role in determining whether a word will be produced correctly or not. Low neighborhood density may serve as predictive information for the monitoring system to signal the likelihood of an upcoming error. In this sense, the P2 amplitude difference could reflect a predictive neurophysiological marker of subsequent error production. The benefit of such a predictive mechanism would be to trigger adjustments in attentional control aimed at prevention, or repair of the error before articulation is fully planned. However, these covert repairs would lead to correct responses due to early anticipation of the upcoming error (Garnsey and Dell, 1984; Postma and Kolk, 1993; Postma, 2000). The current data cannot specifically support this theory as lower orthographic neighborhood density was associated with larger P2 amplitude and increased error rates in speech production. It remains possible that some trials containing low neighborhood density words did lead to successful repair prior to articulation in the current study, but future experiments will need to be designed to explore this potential link further.

The current response-locked analysis revealed an ERN of typical topography and latency following self-produced speech errors that is consistent with previous neurophysiological findings during speech monitoring tasks (Masaki et al., 2001; Ganushchak and Schiller, 2006, 2008b; Sebastian-Gallés et al., 2006; Möller et al., 2007). The current study also confirms previous observations of an ERN following overt vocal errors (Ganushchak and Schiller, 2008a; Riès et al., 2011), and extends those findings to a task other than the picture-naming task. As such, the findings presented here contribute to current models of speech production by demonstrating the generalizability of the recruitment of error/conflict monitoring processes supported by the ACC.

Overall, our data support previous suggestions that speech errors are detected by a general conflict monitoring mechanism supported by the ACC that is involved during manual actions (Ganushchak and Schiller, 2008a). Although the neural generator of the ERN observed in speech production tasks has yet to be specified, the neural generator of the ERN has been repeatedly localized to the ACC of the medial frontal lobes in a variety of manual response tasks (see Holroyd and Coles, 2002 for review). Given that the ERN observed in the current study was of typical timing and topography to those observed during manual action tasks, it is reasonable to postulate that the ACC also plays a role in monitoring speech errors. In the current study, the ERN peaked ~75 ms after response initiation. Thus, it is reasonable to assume that the errors occurred during some stage of speech formulation prior to the start of articulation. Under the speech production model presented in Figure 1 of Postma (2000), it is likely that errors in the current study occurred either during inner speech, at the phonological encoding stage, or during formulation of the phonetic plan, after phonological encoding is complete. Either way it is clear that the errors were not detected via the auditory loop. As such, the data are only consistent with the perceptual loop theory (Levelt, 1983; Levelt et al., 1999) if the errors occurred during inner speech (to be detected via the inner loop) but not if the errors occurred during phonetic planning. Designing experiments to further specify the timing of the error will be critical for additional refinement of speech error monitoring models.

In summary, the current data provide support that speech errors can be detected during early speech formulation processes, prior to articulation. The production of speech errors was preceded by increased amplitude in the stimulus-related P2 component. The current data also support the idea that speech errors are monitored by a central error-monitoring system responsible for the detection of other types of action errors. Both correct and incorrect responses showed evidence of pre-articulatory conflict monitoring in the form of the N2 component, and speech errors were associated with an ERN over fronto-central electrode sites. Although the current findings cannot provide definitive support for a specific functional role of error processing during speech formulation, they provide motivation to further explore the potential of predictive, and corrective pre-articulatory mechanisms in speech production.

### Conflict of interest statement

The authors declare that the research was conducted in the absence of any commercial or financial relationships that could be construed as a potential conflict of interest.

## Footnotes

^1^Specifying the onset of a vocal response is not trivial, but it has been done successfully in the past (e.g.,Ganushchak and Schiller, 2008a; Riès et al., 2011). The detected start position in a response can vary when using intensity-based voice-key triggers, such as the one employed here, depending on whether the initial sound in a word is voiced or not. Note that this would result in some jitter in the peak latency of the individual ERP trials making up the response-locked average; thus, it is possible that our ERP waveforms presented below under-represent the true amplitude of the ERN. It is worth noting that this is not an issue for the stimulus-locked waveform averaging since the stimulus triggers did not vary as a function of voiced vs. unvoiced stimuli.

^2^For one participant, only five error trials survived artefact rejection, which is below the minimum of six recommended by Pontifex et al. (2010). The number of error trials for all other participants was seven or great, exceeding the required minimum. To test whether the low number of error trials from this participant affected the results all of the analyses were run a second time with this person removed. The pattern of results, and all significant effects, remained the same. Thus, we chose to leave this person's data in the results section.

## Appendix 1

**Table:**

**List A: b/p substitution stimuli**				
MOPPED	BIG	PAD	BURRED	BEER
RIBBED	PENT	BLANK	BLOTS	PIKES
BUCKS	PINS	PRAISED	SLOPS	BILE
CRAPS	PUFFED	GAB	BLED	POOR
PURSE	PUS	COP	PALE	PORE
PATH	BEST	LOBE	BOUNCED	BOUTS
BRIM	SLAP	PLIGHT	PEGGED	POUND
PANS	BRAY	DIBBED	PIT	BEEN
SUB	NABBED	BAYS	SOB	
PANE	PANG	PALM	PECK	
ROBED	PAR	NIB	BACKS	
BING	LIMBS	BROW	SWABBED	
BILLS	PUTT	PUGS	TRIPE	
CUBS	PATS	PRAWN	PUDGE	
PRIDE	TAB	PONDS	PAILS	
PEACH	BUNS	LABS	PASTE	
PULLS	DRIB	LOPS	BARE	
CABS	BOX	BELTS	PEAKS	
PUNKS	FLAB	KNOB	BEARS	
BETS	BARK	BUSHED	BEAT

**Table:**

**List B: b/p substitution stimuli**				
MOBBED	PIG	BAD	PURRED	PEER
RIPPED	BENT	PLANK	PLOTS	BIKES
PUCKS	BINS	BRAISED	SLOBS	PILE
CRABS	BUFFED	GAP	PLED	BOOR
BURSE	BUS	COB	BALE	BORE
BATH	PEST	LOPE	POUNCED	POUTS
PRIM	SLAB	BLIGHT	BEGGED	BOUND
BANS	PRAY	DIPPED	BIT	PEEN
SUP	NAPPED	PAYS	SOP	
BANE	BANG	BALM	BECK	
ROPED	BAR	NIP	PACKS	
PING	LIMPS	PROW	SWAPPED	
PILLS	BUTT	BUGS	TRIBE	
CUPS	BATS	BRAWN	BUDGE	
BRIDE	TAP	BONDS	BAILS	
BEACH	PUNS	LAPS	BASTE	
BULLS	DRIP	LOBS	PARE	
CAPS	POX	PELTS	BEAKS	
BUNKS	FLAP	KNOP	PEARS	
PETS	PARK	PUSHED	PEAT

**Table:**

**List A: d/t substitution stimuli**				
TRAINS	HOODS	WELTS	MADE	PLOT
NOD	TIME	SIDES	WORT	BUT
DEAR	FATE	SAT	CARTS	BOLD
TRENCHES	WEDS	NODES	FOOD	TRIPS
FONT	TIRE	CLOUT	TILL	TAMP
TEAL	MAD	AND	TENSE	BIDE
GOADS	MOUNTS	SEND	GRIT	TRESSES
KITS	COT	SQUADS	LET	CLOD
DUNES	RODS	GOD	SKITS	TEEM
FEET	GREET	DROOPS	TAME	TRAWL
DINE	ROTE	FROND	GRADES	
CUD	DROLL	DRUNK	DOWN	
SLID	TRILL	FAT	MOOT	
MEAD	TOWELS	TUCKS	COLDS	
HID	DRY	DEN	LENT	
CURT	DOLE	WANDS	DABS	
WARTS	RIDES	GRANT	BUILT	
DUSK	TUG	HAT	TARE	
DELL	MOULD	LID	PODS	
GUILDS	MELDS	CHARD	DUB

**Table:**

**List B: d/t substitution stimuli**				
DRAINS	HOOTS	WELDS	MATE	PLOD
NOT	DIME	SITES	WORD	BUD
TEAR	FADE	SAD	CARDS	BOLT
DRENCHES	WETS	NOTES	FOOT	DRIPS
FOND	DIRE	CLOUD	DILL	DAMP
DEAL	MAT	ANT	DENSE	BITE
GOATS	MOUNDS	SENT	GRID	DRESSES
KIDS	COD	SQUATS	LED	CLOT
TUNES	ROTS	GOT	SKIDS	DEEM
FEED	GREED	TROOPS	DAME	DRAWL
TINE	RODE	FRONT	GRATES	
CUT	TROLL	TRUNK	TOWN	
SLIT	DRILL	FAD	MOOD	
MEAT	DOWELS	DUCKS	COLTS	
HIT	TRY	TEN	LEND	
CURD	TOLE	WANTS	TABS	
WARDS	RITES	GRAND	BUILD	
TUSK	DUG	HAD	DARE	
TELL	MOULT	LIT	POTS	
GUILTS	MELTS	CHART	TUB	
